# Implementing Appetite to Play at scale in British Columbia: Evaluation of a Capacity-Building Intervention to Promote Physical Activity in the Early Years

**DOI:** 10.3390/ijerph17041132

**Published:** 2020-02-11

**Authors:** Kasra Hassani, E. Jean Buckler, Jennifer McConnell-Nzunga, Sana Fakih, Jennifer Scarr, Louise C. Mâsse, Patti-Jean Naylor

**Affiliations:** 1 Child Health BC, Provincial Health Services Authority, Vancouver, BC V6J 4YC, Canada; kasra.hassani@cw.bc.ca (K.H.); jsmcconn@uvic.ca (J.M.-N.); sana.fakih@cw.bc.ca (S.F.); Jennifer.Scarr@cw.bc.ca (J.S.); 2 School of Population and Public Health, Faculty of Medicine, University of British Columbia, Vancouver, BC V6T 1Z3, Canada; jean.buckler@bcchr.ca (E.J.B.); lmasse@bcchr.ubc.ca (L.C.M.); 3 British Columbia Children’s Hospital Research Institute, 4480 Oak St., Vancouver, BC V6H 3N1, Canada; 4 School of Exercise Science, Physical and Health Education, Faculty of Education, University of Victoria, Victoria, BC V8W 3P1, Canada

**Keywords:** public health intervention, scale-up, early years, physical activity, healthy eating

## Abstract

Childcare is a critical target for promoting children’s physical activity (PA) and physical literacy (PL). With emerging evidence about the efficacy of policy and capacity-building strategies, more information about how to bring these strategies to scale is needed. This paper describes implementation at scale of Appetite to Play (ATP), a capacity-building intervention for childcare providers, and examines the implementation and impact on early years providers’ capacity to address PA. The ATP implementation evaluation was a natural experiment that utilized a mixed methods concurrent parallel design framed within the Reach, Effectiveness, Adoption, Implementation, Maintenance framework (RE-AIM). Workshop and website tracking assessed reach and adoption. Surveys and interviews with workshop participants and stakeholders assessed satisfaction, implementation, and maintenance. Training reached 60% of British Columbia municipalities and 2700 early years providers. Significant changes in participants’ knowledge and confidence to promote PA and PL were achieved (*p* > 0.01–0.001). Childcare level implementation facilitators as reported by early years providers included appropriate resources, planning, indoor space, and equipment, whereas weather and space were reported barriers. The stakeholder advisory group viewed the stakeholder network and Active Play policy as facilitators and adjustments to recent shifts in childcare funding and previous initiatives as barriers to implementation. ATP was scalable and impacted provider knowledge, confidence, and intentions. The impact on actual policies and practices, and children’s PA needs to be assessed along with sustainability.

## 1. Introduction

Participation in sufficient physical activity during the early years is an important ingredient in healthy childhood development [1,2,3]. Early years providers, individuals who work in the early childhood education and care sector including family childcare and parent participation programming, represent a key population to target in improving the physical activity behaviours of young children, particularly given evidence that children are insufficiently active while attending childcare programming [4,5]. Early years providers may participate in relatively short (minimum of 8–16 months in a Canadian context) training programs [6], and receive little to no training in physical activity or related constructs [7,8].

Significant research has examined what factors are influential on the physical activity opportunities and behaviours of children in childcare and how best to improve them [9,10,11,12]. There are mixed results about the efficacy of policy implementation [9,10,11,12,13]. Several studies have concluded that when physical activity policies do not result in increased physical activity, it is likely due to a lack of training in and implementation. Training of early years providers in physical activity has demonstrated significant success in increasing physical activity behaviours of children [13,14,15,16]. Based on this body of evidence, the provincial government of British Columbia (BC) enacted a policy outlining a series of physical activity and sedentary behaviour standards, formally entitled the Director of Licensing Standard of Practice for Active Play (Active Play (AP) Standard) that childcare centres are mandated to follow [17]. To support the enactment of this policy, a capacity building initiative for early years providers, entitled Appetite to Play was scaled up. This initiative was developed through consultation with expert stakeholders (provincial and regional health authorities, early childhood education sector, recreation and sport, researchers, content experts), and a provincial needs assessment (social marketing-oriented survey and focus groups with early years providers). The purpose of Appetite to Play is to ensure early years providers have sufficient capacity to implement evidence-based policies, practices, and environments that support physical activity and healthy eating, and help them comply with the AP Standard.

Historically, physical activity interventions take place on a small scale, with limited plans for scale-up or widespread implementation [18]. Appetite to Play is a public health initiative developed for dissemination at scale and is based on existing evidence and resources customized for stakeholder needs rather than a researcher-driven process mobilizing a specific intervention from pilot testing to efficacy, effectiveness, and then scale-up [18]. Ogilvie and colleagues [18] describe this as a practice-based evidence pathway and highlight the need to incorporate these into research evidence. Appetite to Play could also be described as a Type IV scale-up, as it was disseminated at scale immediately after its development without further pilot and efficacy trials [19].

The purpose of this study was to examine the implementation and impact of the Appetite to Play scale-up using the Reach, Effectiveness, Adoption, Implementation, Maintenance (RE-AIM) framework.

## 2. Methods

### 2.1. The Appetite to Play Capacity-Building Intervention

The Appetite to Play initiative incorporated evidence-based recommendations and strategies from the early years field [20], and implementation of science and public health capacity-building literature [21,22,23,24,25,26]. It included content both in physical activity and healthy eating. This paper focuses on the physical activity content of the initiative.

The physical activity content focused on evidence-based recommendations for limiting screen-time to less than 30 min/day, breaking up sitting time, providing 120 min/day of facilitated and unstructured play opportunities and activities that promote fundamental movement skill acquisition, requiring at least 60 min/day of outdoor play, modelling and creating facility level active play and screen-time policies [20]. The capacity-building activities (see Table 1) aligned with key capacity-building strategies outlined in the literature [23,25,26] and Wandersman, Chien and Katz’s [23] Getting to Outcomes Framework.

### 2.2. Scale-up and Implementation Strategy

The scale-up and implementation of Appetite to Play was founded on extensive consultation and ongoing engagement with early years stakeholders across the province. The “innovation” was designed to meet the needs of early years providers and the scale-up based on stakeholder knowledge and recommendations. The development of Appetite to Play and the scale-up strategy was led by a backbone organization Child Health BC and implemented through a partnership with three other not-for-profit organizations with relevant expertise and established partnerships: the YMCA of Greater Vancouver, Childhood Obesity Foundation, and Sport for Life and researchers at the University of Victoria and University of British Columbia. Over time, the British Columbia Recreation and Parks Association became another key partner in delivery. A provincial stakeholder advisory group was set up to advise the partnership group on the development of both the resource, scale-up strategy, implementation (course correction), and to create a framework for sustainability.

A “train the trainer” model was used to spread the capacity-building intervention across the province [24,27]. Master trainers (experts in physical and food literacy, with a minimum of an undergraduate degree and substantive field experience delivering workshops) were trained in the specific content for the Appetite to Play training workshops. The training workshop for both master and regional trainers was developed by a group of physical literacy and nutrition experts with extensive experience in the early years settings, and with relevant physical and nutrition policies, practices and activities. Individuals that were planning and operationalizing the dissemination and training components (e.g., who could describe how regional trainers would register participants, how they would advertise the workshop, how to implement the evaluation components, etc.) were also involved in development and delivery of the pragmatic aspects of the master trainer workshop. Specifically, the physical activity/physical literacy component was delivered by a PhD expert that had been previously involved in disseminating an early years initiative province wide (Healthy Opportunities for Pre-schoolers) [24]. The nutrition consultant was a qualified public health nutritionist with the appropriate experiences with feeding practices, food literacy, and had experience writing training curriculum. Master trainers in turn trained regional trainers from various geographic communities across the province (*n* = 88). Regional trainers had to have a relevant diploma or degree in early childhood education, nutrition/dietetics, kinesiology, recreation, education, adult education, public health, or relevant fields. Two refresher training sessions were held for the regional trainers, one online and one in-person (participants were able to select in-person or online depending on ability to travel to the location). Regional trainers who asked for additional support were connected with master trainers. In addition, the workshop coordinator would review the post-workshop survey results to see if there was a bad review, in which case, they or one of the master trainers would follow up. Finally, a regular newsletter and cross-site sharing was put in place to support regional trainers. The regional trainers then delivered in-person workshops in their respective communities. In-person training was primarily hosted in partnership with BC’s Child Care Resource and Referral Centres (CCRRs). CCRRs offer workshops and training to support quality child care and programming in the early years field in every community across BC. Embedding the training into this existing training infrastructure was intentional to support sustainability. In-person training was also supported through other organizations that provided childcare and early years programming, such as Strong Start, the YMCA, and municipal recreation centres.

### 2.3. Evaluation Design

The Appetite to Play evaluation represents a natural experiment of a primordial prevention initiative [18]; it utilized a mixed methods convergent parallel design with quantitative and qualitative data weighted equally and data from both collection methods were used to generate summary findings when available [28]. Appetite to Play’s evaluation plan was consultative and was designed together with its implementation strategy. It was informed by the implementation evaluation literature [29] and framed for stakeholders using the RE-AIM framework [30]. The framework is intended for the evaluation of community-based interventions and has the capacity to measure outcomes at the staff level, which was necessary for our evaluation [30]. Additional factors and dimensions such as satisfaction and context were incorporated. As well, we modified the dimension of maintenance to “potential maintenance and sustainability”, as significant data was collected and analysed to determine how to facilitate maintenance and sustainability plan, but not enough time has passed to assess maintenance/ sustainability. The dimensions of Appetite to Play’s evaluation and sources of data are summarized in Table 2. We followed the Standards for Reporting Implementation Studies (StaRI) checklist to provide specific study details [31,32]. The completed checklist is included in Supplementary Table S2. The evaluation period was from September 2017 to March 2019, inclusive. No sample size was calculated, the goal was to scale-up the intervention broadly (dissemination), and the implementation goal was to conduct *n* = 225 in-person workshops and reach *n* = 3000 early years providers during that time period. An additional goal was to reach an additional *n* = 1000 early years providers through the e-learning modules. Early in implementation, the goals were adjusted to *n* = 200 in-person workshops and *n* = 10 live online workshops were added. 

Appetite to Play content focused on physical activity and healthy eating behaviours in the early years setting. In person workshops and live online workshops spent half of the time on each of these content areas. For the purpose of this scale-up evaluation, only physical activity data are discussed.

### 2.4. Data Collection

Pre/post face-to-face workshop survey data were collected using anonymous linked paper-based questionnaires. Pre/post survey data for live-online workshops and e-learning modules were collected anonymously using REDCap electronic data capture tools (REDCap, Nashville, TN, USA) hosted at BC Children’s Hospital Research Institute. REDCap (Research Electronic Data Capture) is a secure, web-based software platform designed to support data capture for research studies [33,34]. Surveys collected information about participant demographics (age, experience, role, previous training), knowledge, and confidence scales (5-point Likert scale) related specifically to key workshop content and messages. Questions were designed specifically to measure knowledge, confidence, and intentions related to workshop content and based on efficacy items from other behaviour change tools, and those implemented in a past training initiative [24,35,36]. The post-workshop survey also measured sense of access to implementation resources and “intention to promote” as a construct based on previous work by Rhodes and colleagues [35,36] and the 24 h movement guidelines [20]. It was three Likert-scale statements on determination, motivation, and intent, namely: “I am determined to promote physical activity & physical literacy for children in my care over the next 2 weeks”, “I am motivated to promote physical activity and physical literacy for children in my care over the next 2 weeks", and “I intend to provide activities that limit sedentary behaviours for children in my care over the next 2 weeks." McDonald’s omega was high (>0.8) for the construct in all modalities, thus indicating its reliability.

Qualitative data were drawn from three sources: interviews and/or focus groups with (1) stakeholders and (2) master and regional trainers, and written responses to two open-ended questions on post workshop surveys by (3) workshop participants. Focus groups and interviews were carried out by members of the research team (JMN, EJB, PJN), with training in qualitative methodology. Interviews and focus groups were conducted in-person or over the phone and were recorded and transcribed verbatim. Written responses were manually entered into a spreadsheet verbatim. Qualitative interviews with childcare providers, stakeholders, and trainers lasted between 20 and 60 minutes. Focus groups lasted approximately 45 minutes. Stakeholder advisory group members were asked questions regarding their role in the capacity building initiative, factors that would facilitate or inhibit the capacity building initiative, and how best to disseminate and promote uptake of the capacity building initiative. Master and regional trainers were asked questions regarding the spread, and response to the capacity building initiative, and how to ensure long-term success of the program. Data from master and regional trainer interviews were analysed together, in order to prevent identification of the master trainers (*n* = 2).

Website analytics data were collected using Matomo freeware [37].

### 2.5. Data Analysis

Paired completed questionnaires were scanned and data were digitized using Remark Office OMR (Gravic, Inc., Malvern, PA, USA). Data were analysed using a mixed-methods approach [28], where conclusions were drawn from both qualitative and quantitative data when available. 

#### 2.5.1. Quantitative Data Analysis

Statistical analyses were performed using RStudio (RStudio, Boston, MA, USA) [38,39] and Microsoft Excel. Factor analyses were done using R Package “Psych” [40]. We used descriptive statistics to describe demographic characteristics and satisfaction of training participants. We performed Wilcoxon signed rank tests to compare the change in average self-assessed knowledge, confidence, and sense of access to resources of workshop participants before and after the training to assess training effectiveness. We used Kruskal-Wallis rank tests to compare the amounts of change (deltas) in between training types to assess their equivalence.

We calculated McDonald’s omega to measure construct reliability for “intention to promote” in both physical activity and healthy eating. The “intention to promote” construct was built on responses to three Likert-scale statements on determination, motivation, and intent, namely: “I am determined to promote physical activity & physical literacy for children in my care over the next 2 weeks”, “I am motivated to promote physical activity and physical literacy for children in my care over the next 2 weeks", and “I intend to provide activities that limit sedentary behaviours for children in my care over the next 2 weeks." The intention construct was built based on previous work by Rhodes and colleagues [35,36] and the 24 h movement guidelines [20]. 

#### 2.5.2. Qualitative Data Analysis

Focus group and interview qualitative data were analysed in NVivo 12 (QSR International, Melbourne, Australia). Thematic analysis was used to analyse the qualitative data using Braun and Clarke’s (2006) 6 steps of thematic analysis. Whenever possible, inductive analysis was utilised; however, due to the nature of the evaluation process, deductive analysis was used in several instances. Written responses were analysed through reading of responses, and frequency counts were created in R. Words less than 10 times and stop words were excluded from analysis. Frequently used words were paired with knowledge of responses from reading and re-reading participant responses to generate response categories.

### 2.6. Ethics Statement

This study was approved by University of British Columbia (H18-01434) and University of Victoria (H18-00666) Research Ethics Boards.

## 3. Results

### 3.1. Reach

#### 3.1.1. Website Reach

During the initiative’s implementation phase (from September 2017 to March 2019), the website had a total number of 25,867 visits (96,804 page views), 10% of which were from individuals in BC, and 38% of which belonged to returning visitors. On average, visitors spent 3 min 56 s on the website and performed 4.5 actions (e.g., page clicks, downloads). These values for returning visitors were 5 min 13 s and 5.1, respectively, indicating higher engagement of returning visitors. Website traffic gradually increased over time. The website was accessed mostly by desktop computers, although usage of smartphones increased over time. Due to limitations in website traffic tracking, we were unable to determine what proportion of the visitors constituted the intervention’s intended audience.

#### 3.1.2. Training Reach and Adoption

Two master trainers were trained and in turn trained 88 regional trainers from across the province through seven regional trainer workshops. Of the 88 trained, 56 (64%) were active and delivered at least one in-person workshop (mean delivery 2.8 workshops (SD 2.6); range 1–11). The regional trainers delivered a total of 195 in-person workshops, training a total of 2328 participants. Of those 2328 participants, 1519 participants (65.2%) provided consent to participate in the research evaluation and completed pre- and post-workshop surveys. The workshops took place in 72 municipalities; however, some individuals travelled to workshops expanding the reach to at least 97 BC municipalities, out of 162 total [41]. A total of 10 live online workshops were held. The online workshops trained a total of 164 participants from 42 municipalities, within and beyond BC. Of the 164 participants, 155 (94.5%) provided consent and completed the surveys. Finally, 270 completed the Physical Literacy e-learning module, of which 145 (53.7%) provided consent and completed the surveys. In summary, a total of 2762 of early years providers were trained through the initiative’s different training modalities, over 95% of which were from BC.

#### 3.1.3. Demographic Profile of Training Participants

Table 3 summarizes the demographic profile for each training modality. The demographics of participants of the different training modalities were fairly similar, a majority identified as female, with an average age near 40 and about 10 years of experience in the early years setting (Table 3). Participant age and experience, however, ranged widely. About two-thirds identified as an early childhood educator. Early childhood educators are certified by the provincial government to be employed in licensed early years facilities, but may work in a variety of fields. Early childhood educators are a subtype of early years providers, which includes individuals working in conjunction with an early childhood educator in a licensed program or in license not required or parent participant centres where certification is not mandated. The majority of participant workplaces included preschools and group childcares, followed by licensed family-based, parent participation, recreational, and other programs.

### 3.2. Training Satisfaction and Effectiveness

#### 3.2.1. Training Satisfaction

Participants were overall highly satisfied with different aspects of the training, including content, novelty, delivery, and usefulness for all training modalities (Table 4) as measured through post-training surveys. We did not see any significant difference between modalities in proportions of participants who were “satisfied” or “highly satisfied” with the training in any of the aspects (all Chi-squared tests, *p* > 0.05).

The ratings for novelty of the training content was lower than the levels of satisfaction.

#### 3.2.2. Training Effectiveness

Our results show that all training modalities significantly improved the knowledge and confidence of the participants (Table 5). Except for a few aspects, we saw no significant difference between the three training modalities in changes to knowledge and confidence of the training participants (Supplementary File 1).

#### 3.2.3. Post-Training Intentions

We found intentions to promote physical activity following the workshop to be exceptionally high among training participants and not significantly different across training modalities, pointing to their equivalence (Kruskal–Wallis chi-squared = 4.3484, df = 2, *p*-value = 0.1137) (Table 6).

### 3.3. Implementation

#### 3.3.1. Workshop Participants

Six categories of implementation facilitators emerged from participant responses and two categories of barriers emerged. These are outlined with supporting quotes in Table 7.

#### 3.3.2. Master and Regional Trainers

Two major categories of feedback emerged from the master and regional trainer interviews. First, they highlighted the positive response from the participants and that the information provided made an impact. In particular, they highlighted the reaction of early years providers to data showing low physical activity levels in early years’ setting. “People were shocked about the lack of physical activity in childcare” (regional trainer 2).

Second, they highlighted some context elements as facilitators or barriers. Regional trainers viewed childcare resource and referral centres as facilitating implementation of the workshops “Because its {early years providers}… partnering with CCRRs, I organize it, state date and time and the CCRR advertises and sends out through networks. Brings it up at meetings” (regional trainer 6). As well, they reported that the personal connections of the individual regional trainers enhanced their capacity to promote the workshop. Conversely, in rural areas recruitment and spreading the word presented challenges, particularly in very small communities without any “real local hub”.

#### 3.3.3. Stakeholder Advisory Group

Stakeholders highlighted that both within the stakeholder group itself, and within their own communities, regions, and networks they were an active leader in promoting the Appetite to Play program; an implementation facilitator.

“I think it’s just been a really successful partnership with all of the groups that have been involved with promoting the program and it’s just really … lended to the collaboration”

(Stakeholder 9)

“I see our role … as information sharing and also network connection sharing. So, I think it’s mostly networking for our part with a little bit … a guidance role being a part of the advisory committee”

(Stakeholder 5)

Stakeholders identified two major challenges in the reach and uptake of Appetite to Play. The first theme was “Too much to do, too little time”. Provincial government increases in funding for the early years, which was seen as good news, resulted in significantly more administrative work for early years providers.

“People are just so busy. And there is a bit of a … crisis out there with childcare and then with all the new initiatives, provincial initiatives it takes up a lot of people’s … time”

(Stakeholder 1)

The second theme that emerged from the data was “been there done that”. Stakeholders felt there was likely a perception that the materials were a repeat of past initiatives and in a resource constrained situation, with the multi-level changes occurring in childcare as a result of provincial government funding changes this influenced the time they were willing to commit.

“They had Food Flair, and then they had … HOP (Healthy Opportunities for Preschoolers) and then they had had Healthy Beginnings and so I think there was a little bit of like ‘another program?’ Like ‘we just got trained up on the previous ones and have delivered those.’”

(Stakeholder 3)

### 3.4. Potential Maintenance and Sustainability

Regional trainers felt the reach of Appetite to Play could be far bigger than what it was currently and had a number of recommendations for expanding the reach of Appetite to Play: booking workshops far in advance, connecting with local libraries and public health dietitians, increasing the use of social media to promote the program and engage the early years’ community, and incorporating actionable activities into the newsletters that would engage and continue to keep early years providers connected with the program.

Two major recommendations for continued uptake of the program came from the stakeholders:

One, the use of multi-modal training programs (i.e., online, in person) exemplified by one participant:

“the reality is, is that this is the way people learn nowadays and it’s important to them to be able to fit it in, you know to their life, so having that online version is really important I think to the future sustainability”

(Stakeholder 1)

Two, the potential of using current success as a model to extend the reach of the program.

“So, like if in promotional materials, it’s like ‘2000 of BC people have already taken this training like are you missing out?’ … That that might help to kind of pique people’s interests.”

(Stakeholder 7)

## 4. Discussion

Scale-up of efficacious public health interventions to achieve population health impact has been highlighted as a public health and research priority [30,42,43]. This priority, coupled with a call for more practice-based evidence to inform scale-up [18,44] indicates a need for research to shift focus from small-scale intervention to large scale implementation science initiatives. With the early years as a critical and influential time period for establishing physical activity and its related developmental benefits plus a fairly robust evidence-base on effectiveness of action in the childcare setting, the BC government enacted an active play policy and a capacity-building intervention to support the policy implementation [17]. This provided our team with a relatively infrequent opportunity [45,46] to examine the implementation and impact of an early years’ capacity-building intervention (Appetite to Play scale-up) to increase physical activity and physical literacy in childcare settings using the RE-AIM framework [30].

Reach and adoption was relatively high, covering over half of BC municipalities with 215 in-person and live online workshops plus training-learning modules reaching ~2700 individuals over 18 months. Two thirds of those were certified early childhood educators (representing 11% of educators in BC), and the remaining were assistant or support professionals that served the early years sector (e.g., licensing officers, public health, etc.) and professionals serving children in different spaces (e.g., recreation). For comparison, the first provincial training for Healthy Opportunities for Preschoolers in British Columbia utilized 15 regional trainers and delivered 43 workshops (*n* = 430 childcare providers) in 9 months (Naylor and Temple, 2013). During a further scale-up of HOP in the LEAPBC initiative (unpublished data), 78 workshop leaders (57 actively delivering and 35 delivering 2+ workshops) delivered 78 HOP workshops in 9 months reaching 775 early years providers or community stakeholders. Romp and Chomp, an Australian community-based obesity prevention intervention took place between 2004 and 2008 and collected data at the childcare centre and child level. Eight large centres, 76 home-based care providers, and 45 preschools were targeted, with 2850 children participating in the data collection and an estimated 12,000 children targeted by the intervention [47].

As a further comparison, the Action Schools! BC Support Team and Trainers (*n* = 75 active) delivered 400–500 school-based workshops per year over a ten-year window and after 10 years of implementation, the initiative was active in 100% of BC school districts and over 90% of schools had registered [48]. In 2006, a student leadership component was added and over 1300 student PA leadership workshops were provided over 8 years (approximately 162/year; unpublished data). In the US, a health-related PE program (SPARK) was evaluated for sustainability [49]. Two hundred and seventy-seven schools from seven states adopted SPARK more than a year before their sustainability evaluation [49]. All of these examples are comparable with the regionalized delivery of ATP. Beyond this, website traffic was high; ~10% of traffic were BC participants and significant returning visitors, and returning visitors spent longer periods of time on the website. This indicates that the content was engaging and useful for visitors [50,51].

Overall, regional trainer retention was moderate. The majority of regional trainers facilitated at least one workshop in 18 months, similar to previous research [24]. However, data were not collected as to why the remaining trainers did not facilitate any workshops. A similar study using a train the trainer model found that trainers did not facilitate workshops due to personal reasons, e.g., illness, transition to a new job, and work responsibilities [24]. Future studies should consider tracking the trainer’s reasons for not facilitating any workshops given the resources that go into providing training.

Satisfaction and effectiveness of Appetite to Play at the level of the early years provider was high with significant improvements in knowledge and confidence in all areas of the training for participants attending in person and live online workshops, and almost all areas for participants who completed the physical literacy e-learning module. It is possible that where the e-learning module did not reach significance were due to the lack of a question and answer period in the e-learning module (i.e., an opportunity to speak to a workshop facilitator regarding unclear information provided) and/or relatively smaller sample size. Following completion of the workshop, intentions to promote physical activity were high. These findings are in line with previous literature that indicated that early years provider trainees were significantly more confident in their capacity to facilitate physical activity when they had participated in training in physical activity and/or physical education [8].

Appetite to Play implementation at the early years provider level was reportedly challenged by environmental factors (e.g., space, weather), facilitated by organizational supports (equipment and indoor space), and resources (e.g., planning, access to websites/books). These barriers and facilitators are in line with existing early years research [9,10,52]. Notably, Appetite to Play was frequently cited as a facilitator to implementing physical activity and physical literacy. In regards to the context, uptake may have been limited due to similar early years training that had been available provincially over the previous decade (e.g., Healthy Opportunities for Preschoolers, Healthy Beginnings). This was demonstrated in the quantitative data, where few participants indicated that the workshop content was new, and echoed by stakeholders, who highlighted the multiple initiatives that had occurred provincially as an implementation barrier. Beyond this, the implementation of the Active Play Standards likely influenced uptake, as licensed childcare facilities were now required to adhere to specific physical activity policies. This, as noted in the workshop participant data, was where several early providers indicated the new standards as a contextual facilitator in implementing physical activity. Stakeholders further highlighted the positive influence of the network of stakeholders engaged in Appetite to Play and the negative influence of significant turbulence in the childcare system (new funding plus associated administrative requirements). A significant maintenance plan was developed using recommendations from our preliminary results that were distributed to government stakeholders, and this plan has since been put into place for Appetite to Play. Both care providers and stakeholders were optimistic about the potential of maintenance and sustainability and offered suggestions, but long-term sustainability remains to be seen.

When comparing to the literature and through the lens of our data, the 18-month implementation of Appetite to Play appears to have been successful. Reach, regional adoption, and stakeholder satisfaction were high and comparable to other scale-ups. It appeared both implementable and efficacious; facilitating increased knowledge, confidence, and implementation of physical activity and physical literacy activities. While the evaluation of this scale-up was pragmatically constrained and does not provide child-level data or direct childcare level measures, there are several indicators that point to the potential of the scale-up for improving child-level physical activity and physical literacy. High intentions to promote physical activity are promising, as research indicates that the provision of opportunity for physical activity in childcare is associated with higher levels of physical activity [9]. As well, higher confidence to model physical activities will likely translate into higher physical activity, as educator presence (e.g., prompting) is associated with higher engagement in physical activity in childcare [13]. In addition, increased care provider knowledge and confidence in promoting locomotor, manipulative, balance, and stability skills may translate to providing opportunities to develop fundamental movement skills, a component of physical literacy [53]. Children with stronger fundamental movement skills are more likely to participate in lifelong physical activity [54]. Beyond this, Appetite to Play improved early years providers’ confidence in improving children’s motivation to move, and confidence in movement skills, key psychological components of physical literacy.

### Limitations and Strengths

This study should be viewed in light of its limitations. As a natural experiment, there was no comparison condition to evaluate if the shifts in knowledge and confidence were directly attributable to the training. Further the primary aim of the study was pragmatic, to evaluate the scale-up of Appetite to Play, thus, data were collected at the level of stakeholders, regional trainers, and early years providers, but not at the facility or child level. Therefore, we do not have data to verify if the increases in knowledge and confidence at the early years provider level translate to changes in facility level policy and practices, or physical activity and physical literacy at the child level. Knowledge and confidence measures were designed pragmatically to evaluate key messages and were not previously validated. Satisfaction data should be interpreted with caution, as in person and live online workshop responses for satisfaction with workshop content and delivery addressed both the healthy eating and physical activity components. However, there was no difference between reported satisfaction for any of the training modalities including the e-learning modules where healthy eating and physical activity were presented separately. Additionally, the healthy eating knowledge and confidence changes were similar and we have included the data in Supplementary File 1. This study was not registered with a recognized study registration body which could be viewed as a reporting bias. Two components of the StaRI checklist were not met in full. No economic evaluation was undertaken, and fidelity to intervention was not tracked, with the exception of review of trainer feedback from participants. Website analytics data do not distinguish between intended audience and others members of the public who accessed the website. Finally, the intervention incorporated a set of better practices from the literature, rather than following a traditional scientific pathway from a fully piloted intervention package through to scale-up.

Conversely, the study also has significant strengths. The intervention was designed with the real world needs of stakeholders front and centre and is based on current evidence. Additionally, the evaluation was based on a large sample size of over 1500 consented participants. As well, data to examine the scale-up were drawn from multiple sources (stakeholders, regional trainers, early years providers) with multiple modalities (surveys, open ended text responses, interviews, and focus groups). Finally, data were collected in line with empirically evidenced frameworks: implementation evaluation literature and the RE-AIM framework [18,23,30,45].

## 5. Conclusions

This paper demonstrates that an evidence-based capacity-building intervention can improve early years providers’ knowledge, confidence, and intention to promote physical activity and physical literacy. This is a necessary step in changing the policies, practices and environments that influence child physical activity behaviour in the early years. While the relevant evidence suggests Appetite to Play is likely to have positive impacts on the child-level health behaviours while attending childcare, research is needed to verify this and is currently underway in British Columbia. Implementation was supported by provincial stakeholders and at-the-finger tips resources, planning, equipment, and indoor space. It was also challenged by a rapidly changing provincial funding and programming context and at the facility level, weather and lack of space. Efforts to support providers in developing strategies and contingency plans to overcome these modifiable and non-modifiable challenges are warranted.

## Figures and Tables

**Table 1:** Key capacity-building intervention components for early years providers.

Intervention Component	Description
**Training**	(1) 3 h in-person workshops eligible for 3 professional development (PD) credits (1.5 h dedicated to physical activity and physical literacy), (2) 3 h live-online workshops delivered via GoToMeeting (LogMeIn Inc, Boston, MA, USA) eligible for 3 PD credits similar to in-person workshops, and (3) self-paced e-learning modules in physical literacy and healthy eating, each eligible for 1.5 h of PD credits.
**Toolkit (tools)**	Website based early years provider “toolkit” (www.appetitetoplay.com) The website, updated weekly, houses (1) a set of interactive tools to assist early years providers in program planning (e.g., weekly physical activity and meal planners, and self-assessment/audit tools), (2) a set of recommended practices on physical activity in the early years, linked to the Active Play Standard (AP Standard), (3) active play ideas, tips and direction, (4) access to the e-learning modules, and (5) registration for online workshops
**Technical support**	Regular new content including new tips and activity ideas via social media, e-newsletters, and website updates; email and telephone support for users inquiring and registering for training
**Community of practice**	A community of practice was piloted in collaboration with British Columbia (BC) Early Years Professional Development (EYPD) portal. EYPD is a hub for early years professionals to find and post training events for PD. The forum was moderated by regional trainers and monitored by the implementation team and master trainers.
**Marketing and communications**	Postcards, brochures, branded giveaways and a promotional video were created and used to promote Appetite to Play to early years providers across the province. Licensing officers promoted the training and resources to early years providers as a tool that can be used to meet licensing requirements. Weekly social media posts and bimonthly e-newsletters were also used to promote the initiative.

**Table 2:** Appetite to Play’s Evaluation Matrix.

Dimension	Indicators/Questions	Sources of Data
**Reach**	The total number of early years providers who accessed the initiative	Administrative data on training Website traffic
**Effectiveness**	The ability of the initiative’s trainings to increase knowledge, confidence, and intentions of early years providers in physical activity and healthy eating	Pre/post training surveys
**Adoption**	The number of and location of training workshops offered across BC	Administrative data
**Implementation**	Successes and challenges of implementation of different components of Appetite to Play	Administrative data Partner communication, focus group or individual interviews with stakeholder advisory committee, master trainer and regional trainers Post training surveys from in person workshops
**Context**	Contextual factors that impacted the intervention’s implementation and uptake	Interviews with stakeholder advisory group and regional and master trainers Post training surveys from in person workshops
**Potential Maintenance and Sustainability**	The sustainability of Appetite to Play beyond its Phase 2 Implementation Long-term use of Appetite to Play learnings and resources in early years settings Piloting of live online workshops, researching equivalence of training modalities for lower cost delivery of training.	Follow-up interviews with master trainers, and regional trainers about potential for long-term use Pre/post training surveys of live online workshops and e-learning modules
**Satisfaction**	The satisfaction training early years providers had with the content, and delivery	Pre/post training surveys

**Table 3:** Summary demographics of participants of different training modalities.

Training Modality	In-Person Workshop	Live Online Workshop	E-Learning Module
Number of respondents (response rate)	1519 (65.2%)	155 (94.6%)	145 (53.7%)
Percent female	95.7%	96.7%	96.2%
Mean age (SD)	39.7 (12.3)	42.9 (11.4)	38.9 (11.2)
Mean years of experience in the early years (SD)	11.7 (10.0)	13.2 (10.1)	11.1 (10.0)
Percent early childhood educator	61.8%	76.8%	64.6%
Percentage without any prior physical activity training	39.9%	50.9%	50.3%

SD: Standard deviation.

**Table 4:** Overall satisfaction of participants with different training modalities.

Training Modality	In-Person Workshop	Live Online Workshop	E-Learning Module
Workshop content was new to me	2.9 (1.3)	2.7 (1.1)	3.1 (1)
Overall satisfied with content	4.2 (1.1)	4.3 (0.6)	4.3 (0.6)
Overall satisfied with delivery	4.2 (1.2)	4.4 (0.6)	4.3 (0.7)
Training will be useful	4.1 (1.2)	4.1 (0.7)	4.2 (0.7)

Numbers present mean and standard deviation on a digital scale from 1 to 5, 1 being the lowest and 5 the highest on the scale, converted from responses to 5-item Likert scale questions.

**Table 5:** Perceived knowledge and confidence in different aspects of physical activity before and after training for different training modalities.

	Training Modality	In-Person Workshops			Live Online Workshops			E-Learning Module		
Field		Pre-Survey Mean (SD)	Post-Survey Mean (SD)	*p*-Value	Pre-Survey Mean (SD)	Post-Survey Mean (SD)	*p*-Value	Pre-Survey Mean (SD)	Post-Survey Mean (SD)	*p*-Value
Knowledge	Locomotor skills	3.1 (1)	4 (0.7)	***	3.1 (1)	3.8 (0.8)	***	3.4 (0.8)	4.1 (0.8)	**
	Manipulative skills	3.3 (1)	4 (0.7)	***	3.3 (1)	3.8 (0.8)	***	3.6 (0.9)	4.1 (0.7)	**
	Balance and stability activities	3.4 (0.9)	4.1 (0.7)	***	3.3 (0.8)	4 (0.7)	***	3.7 (0.8)	4.2 (0.7)	*
	Moderate to vigorous physical activity	3.4 (0.9)	4.1 (0.7)	***	3.4 (0.9)	3.9 (0.7)	***	3.7 (0.8)	4.1 (0.7)	**
	Short burst, intermittent activity	3.1 (1)	4 (0.8)	***	3.2 (0.9)	3.7 (0.8)	***	NA	NA	
	Facilitated physical activities	3.4 (0.9)	4.1 (0.7)	***	3.4 (0.8)	3.9 (0.7)	***	3.7 (0.8)	4.1 (0.7)	*
	Physical literacy	3.1 (1)	4.1 (0.7)	***	3.1 (0.9)	3.9 (0.7)	***	3.4 (0.9)	4.2 (0.7)	**
	Providing opportunities for exploration and free play	3.9 (0.9)	4.3 (0.7)	***	3.9 (0.9)	4.1 (0.7)	*	3.9 (0.8)	4.2 (0.7)	*
	Adapting physical activities for different ages, abilities, and cultures	3.6 (1)	4.1 (0.8)	***	3.5 (0.9)	3.9 (0.7)	***	3.7 (0.9)	4.1 (0.7)	*
	Creating an environment that encourages physical activity	3.8 (0.9)	4.2 (0.7)	***	3.6 (0.9)	4 (0.7)	***	3.9 (0.9)	4.1 (0.7)	NS
	Limiting sedentary behaviours (e.g., screen time and prolonged sitting)	3.7 (1)	4.2 (0.8)	***	3.8 (1)	4.1 (0.7)	**	4 (0.8)	4.3 (0.7)	NS
	Communicating about physical activity/physical literacy with families	3.1 (1)	3.9 (0.8)	***	3.2 (0.9)	3.9 (0.8)	***	3.8 (0.9)	4.2 (0.7)	*
	Developing organizational policies for physical activity/active play	3 (1.1)	3.8 (0.9)	***	3.2 (1)	3.9 (0.9)	***	3.5 (1)	4 (0.8)	**
	Developing organizational policies for limiting screen time	3 (1.1)	3.8 (0.9)	***	3.3 (1.1)	3.9 (0.9)	***	3.6 (1.1)	4.1 (0.9)	**
Confidence	Locomotor skills	3.2 (1)	4.1 (0.7)	***	3.3 (1)	3.9 (0.7)	***	3.7 (0.8)	4.2 (0.7)	**
	Manipulative skills	3.5 (1)	4.1 (0.8)	***	3.5 (0.9)	4 (0.7)	***	3.8 (0.9)	4.3 (0.7)	**
	Children’s movement confidence	3.6 (0.9)	4.2 (0.7)	***	3.6 (0.9)	4.1 (0.7)	***	3.9 (0.8)	4.3 (0.7)	*
	Children’s motivation to move	3.6 (0.9)	4.2 (0.7)	***	3.7 (0.9)	4 (0.7)	***	3.9 (0.8)	4.3 (0.7)	*
	Balance and stability activities	3.6 (0.9)	4.2 (0.7)	***	3.5 (0.9)	4 (0.7)	***	3.8 (0.8)	4.4 (0.7)	**
	Moderate to vigorous physical activity	3.5 (0.9)	4.2 (0.7)	***	3.5 (0.9)	4 (0.7)	***	3.8 (0.8)	4.3 (0.7)	**
	Frequent short burst, intermittent activity	3.4 (1)	4.1 (0.8)	***	3.4 (1)	4 (0.7)	***	3.8 (0.8)	4.3 (0.7)	**
	Facilitated physical activities	3.6 (0.9)	4.2 (0.7)	***	3.6 (0.9)	4 (0.7)	***	4 (0.9)	4.2 (0.7)	NS
	Provide opportunities for exploration and free play	4 (0.8)	4.3 (0.7)	***	4 (0.9)	4.2 (0.7)	*	4.1 (0.9)	4.3 (0.6)	NS
	Adapt physical activities for different ages, abilities, and cultures	3.8 (0.9)	4.1 (0.7)	***	3.7 (0.9)	4 (0.7)	**	3.7 (0.9)	4.3 (0.7)	**
	Create an environment that encourages physical activity	3.9 (0.9)	4.3 (0.7)	***	3.8 (0.9)	4.2 (0.6)	***	4.1 (0.8)	4.3 (0.7)	NS
	Limit sedentary behaviours (e.g., screen time and prolonged sitting)	3.7 (1)	4.2 (0.7)	***	3.9 (0.9)	4.1 (0.7)	**	4 (0.9)	4.3 (0.6)	*
	Communicate about physical activity/physical literacy with families	3.3 (1)	4 (0.8)	***	3.5 (0.9)	3.9 (0.7)	***	3.7 (0.9)	4.2 (0.7)	*
	Developing organizational policies for physical activity/active play	3.2 (1.1)	3.9 (0.9)	***	3.4 (0.9)	3.9 (0.8)	***	3.6 (1)	4.1 (0.9)	*
	Develop organizational policies for limiting screen time	3.2 (1.1)	3.9 (0.9)	***	3.5 (1)	4.1 (0.8)	***	3.6 (1)	4.2 (0.8)	**
	Model physical activities	3.8 (0.9)	4.3 (0.7)	***	3.7 (0.9)	4.1 (0.7)	***	3.9 (0.8)	4.4 (0.7)	**
Resources	Do you feel you have the resources or tools you need to promote physical activity and physical literacy for children in your program?	2.7 (0.9)	2.1 (0.9)	***	2.6 (0.9)	1.9 (0.7)	***	2.1 (0.9)	1.6 (0.8)	**

Numbers present mean and standard deviation on a digital scale from 1 to 5, 1 being the lowest and 5 the highest on the scale, converted from responses to 5-item Likert scale questions. Wilcoxon Signed Rank Test to compare pre/post values. *p*-value: *: <0.05, **: <0.01, ***: <0.001, NS: Not significant.

**Table 6:** Post-training intention in promoting physical activity.

Training Modality	In-Person Workshop		Live Online Workshop		E-Learning Module	
	Mean (SD)	McDonald’s omega	Mean (SD)	McDonald’s omega	Mean (SD)	McDonald’s omega
Intention to promote physical activity	4.3 (0.6)	0.83	4.3 (0.5)	0.89	4.1 (0.6)	0.89

Numbers present mean and standard deviation on a digital scale from 1 to 5, 1 being the lowest and 5 the highest on the scale, converted from responses to 5-item Likert scale questions.

**Table 7:** Key themes and sample quotes from written responses from in person workshop participants on barriers and facilitators to meeting the physical activity standards.

Facilitators	Barriers
**Availability of Resources** “I think the Appetite to Play site is going to make a very good resource to refer to, to make it easy to implement physical activity in a variety of new games and activities. I look forward to teaching myself so I can further reach the children.”	**Poor weather conditions** “Raining/winter/cold weather. We don’t have an indoor gym.” “Poor air quality (smoke) but we have access to gym most days.”
**Using Resources** “Doing activities such as the rope one we learned today.”	**Insufficient or inadequate space** “Sometimes the centre may be busy and it is harder to create large motor movements, because of limited space.”
**Planning** “I was already practicing these things. New fresh ideas are always welcome. I feel planning more activities would be beneficial, creating a recording system.”	
**Provision of equipment** “Music, a variety of balls, bean bags, scarves, ribbons, parachutes, running … the kids love it when I chase them. Riding toys. Climbing equipment.”	
**Use of indoor spaces** “The fact that we have alotted gym time with various equipment”	
**Active Play policy** “Mandatory outdoor playtime in childcare policy.”	

## Supplementary Materials

The following are available online at https://www.mdpi.com/1660-4601/17/4/1132/s1, Supplementary File 1. Kruskal-Wallis test to see if the changes in knowledge and confidence in physical activity were significantly different between the training modalities (in-person workshops, live online workshops, e-learning module). Supplementary File 2. StaRI checklist.
