# Supplementary material for: Implementing Appetite to Play at scale in British Columbia: Evaluation of a Capacity-Building Intervention to Promote Physical Activity in the Early Years
Source: Int J Environ Res Public Health. 2020 Feb 11;17(4):1132. doi: 10.3390/ijerph17041132 (PMC7068589; doi:10.3390/ijerph17041132)
Supplement: Supplementary file 1 [file ijerph-17-01132-s001.zip › ijerph-688187-supp_table.docx]

Supplementary Data

**Table 1.** Kruskal-Wallis test to see if the changes in knowledge and confidence in physical activity were significantly different between the training modalities (In-person workshops, live online workshops, e-learning module). In majority of areas there was no significant difference between the modalities in the overall amount of change in knowledge and confidence, indicating an overall equivalence of the modalities in terms of effectiveness.

| **Field** | **Area** | **Kruskal-Wallis Chi Square** | **df** | ***p*-value** |
| --- | --- | --- | --- | --- |
| Knowledge | Locomotor skills | 5.3718 | 2 | NS |
|  | Manipulative skills | 3.876 | 2 | NS |
|  | Balance and stability activities | 5.7089 | 2 | NS |
|  | Moderate to vigorous physical activity | 4.8739 | 2 | NS |
|  | Short burst, intermittent activity | 9.5436 | 1 | ** |
|  | Facilitated physical activities | 8.8681 | 2 | * |
|  | Physical literacy | 8.1521 | 2 | * |
|  | Providing opportunities for exploration and free play | 3.4032 | 2 | NS |
|  | Adapting physical activities for different ages, abilities, and cultures | 0.2631 | 2 | NS |
|  | Creating an environment that encourages physical activity | 2.5172 | 2 | NS |
|  | Limiting sedentary behaviors (e.g., screen time and prolonged sitting | 3.2185 | 2 | NS |
|  | Communicating about physical activity/physical literacy with families | 5.0092 | 2 | NS |
|  | Developing organizational policies for physical activity/active play | 5.1281 | 2 | NS |
|  | Developing organizational policies for limiting screen time | 8.5617 | 2 | * |
| Confidence | Locomotor skills | 8.6662 | 2 | * |
|  | Manipulative skills | 2.3572 | 2 | NS |
|  | Children's movement confidence | 3.9026 | 2 | NS |
|  | Children's motivation to move | 6.4399 | 2 | * |
|  | Balance and stability activities | 2.7732 | 2 | NS |
|  | Moderate to vigorous physical activity | 2.5767 | 2 | NS |
|  | Frequent short burst, intermittent activity | 4.4043 | 2 | NS |
|  | Facilitated physical activities | 5.9886 | 2 | NS |
|  | Provide opportunities for exploration and free play | 0.3043 | 2 | NS |
|  | Adapt physical activities for different ages, abilities and cultures | 1.1701 | 2 | NS |
|  | Create an environment that encourages physical activity | 2.2472 | 2 | NS |
|  | Limit sedentary behaviours (e.g., screen time and prolonged sitting | 5.2665 | 2 | NS |
|  | Communicate about physical activity/physical literacy with families | 7.208 | 2 | * |
|  | Developing organizational policies for physical activity/active play | 7.123 | 2 | * |
|  | Develop organizational policies for limiting screen time | 3.0039 | 2 | NS |
|  | Model physical activities | 3.2669 | 2 | NS |
| Resources | Do you feel you have the resources or tools you need to promote physical activity and physical literacy for children in your program? | 0.9881 | 2 | NS |

*p*-value: *: <0.05, **:<0.01, ***:0.001, NS: Not significant***.***
